# A Roadmap of Primary Pandemic Prevention Through Spillover Investigation

**DOI:** 10.3201/eid3108.250442

**Published:** 2025-08

**Authors:** Emily S. Gurley, Raina K. Plowright

**Affiliations:** Johns Hopkins University, Baltimore, Maryland, USA (E.S. Gurley); Cornell University, Ithaca, New York, USA (R.K. Plowright).

**Keywords:** Pandemic, prevention, spillover, investigation, COVID-19, viruses, pandemic preparedness, public health, bioterrorism and preparedness, United States

## Abstract

Since the COVID-19 pandemic, attention and investment in pandemic preparedness have increased. Although there are many valiant plans around pandemic preparedness, they typically involve slowing the spread or mitigating the effects of a pathogen after it has already entered the human population. The task of stopping the pathogen from entering the human population in the first place, spillover prevention, remains a neglected area in discussions and planning for pandemic risk mitigation. Every spillover offers an opportunity to learn about an emerging public health threat and the conditions that aligned to enable spillover occurrence. In this article, we outline One Health approaches for use in spillover investigations, drawing from our experience investigating Hendra and Nipah virus spillovers. We present a roadmap for how findings from those investigations can lead to the development of interventions for spillover and ultimately pandemic prevention.

Pandemics occur when a pathogen is transmitted across continents through human populations that lack prior immunity (*1*). Most pathogens that start pandemics are zoonotic, originating in wildlife or other animals (*1*). Typically, those animal pathogens are novel to humans, so most humans are susceptible, and if those pathogens have or gain the ability to transmit between humans, they pose a pandemic risk. In the wake of the COVID-19 pandemic, pandemic preparedness has been a focus of global engagement. Although such efforts include valiant plans, they largely focus on slowing the spread or mitigating the effects of a pathogen after it has already entered the human population. Initiatives of note include the Coalition for Epidemic Preparedness Innovations plans to deliver vaccines within 100 days of an emerging threat, the World Bank’s investment in surveillance in low- and middle-income countries, and the World Health Organization’s efforts to develop more rigorous global agreements on investigation and collective action. Although those strategies enhance our responses to emerging infection outbreaks, they primarily address scenarios after a pathogen has established transmission between humans. However, the task of stopping the pathogen from entering the human population in the first place, spillover prevention, remains a neglected area in discussions and plans for pandemic risk mitigation.

A spillover occurs when a pathogen infects a new host species (*2*,*3*). The vast majority of spillovers will not lead to an outbreak or pandemic. However, for pathogens with pandemic potential, each spillover into a human is an opportunity to launch a pandemic. Most pandemic prevention plans focus on finding outbreak events earlier, notifying neighboring countries, assembling effective outbreak response teams, and enhancing global surveillance for spillover and outbreak events. Those measures are all crucial. However, preventing the spillover in the first place should be a fundamental component of our global strategy for preventing pandemics.

Numerous initiatives have attempted to identify potential pandemic causing pathogens before they cause outbreaks. One approach is to model geographic areas at high risk for spillovers, correlating putative drivers with locations of past spillovers and overlap of humans and reservoir species (*4*–*6*). Those efforts aim to focus surveillance and resources on areas or species of high risk. Substantial investments have led to the discovery of new viruses infecting rodents, bats, and primates, including viruses that were phylogenetically related to outbreak causing pathogens, suggesting a potential risk for spillover (*7*–*19*). Although such efforts have produced findings of interest, they have not produced actionable public health data. Those approaches do not inform which pathogens are spilling over and the mechanisms driving these events.

Spillovers do provide actionable data. Once an emerging pathogen infects a human, a public health threat is actualized. Those events garner our attention and concern much more than hypothetical risk warnings. Particularly alarming is evidence of transmission of the pathogen from human to human, because this capability is necessary to cause a pandemic. For example, if there was evidence that persons infected with bovine strains of avian influenza H5 across the United States (*20*) were able to infect others, the risk of a pandemic from this virus would increase drastically.

Every spillover offers a critical opportunity to learn about an emerging public health threat and the conditions that aligned to enable the spillover occurrence. Investigating those events requires a transdisciplinary approach, often best conceptualized as a One Health investigation that integrates multiple fields of expertise (Figure 1). The investigation typically begins with medical experts who understand the clinical manifestations of the disease and natural history of infection because the spillover is detected when a sick person seeks care. Spillovers sometimes also occur first in other species, which become bridging hosts to humans. Laboratory analysis of the genetic sequence of the pathogen can provide more information about its origins and potential reservoir hosts. Concurrently, epidemiologic investigations can determine the exposures that led to infection and assess if transmission is ongoing through extensive contact tracing efforts. Next, veterinary and ecologic investigations of animals in the affected communities can identify potential reservoir species and bridging hosts. Social scientists contribute in-depth understanding of how local practices might have enabled exposure and transmission, including human–animal interactions and their drivers. Finally, environmental and ecologic investigations elucidate how changes in the reservoir host condition or distribution might have enabled spillover. The timing of those investigations is critical because the conditions for spillover can be fleeting, so rapid identification and investigation of spillovers is vital.

**Figure 1:**
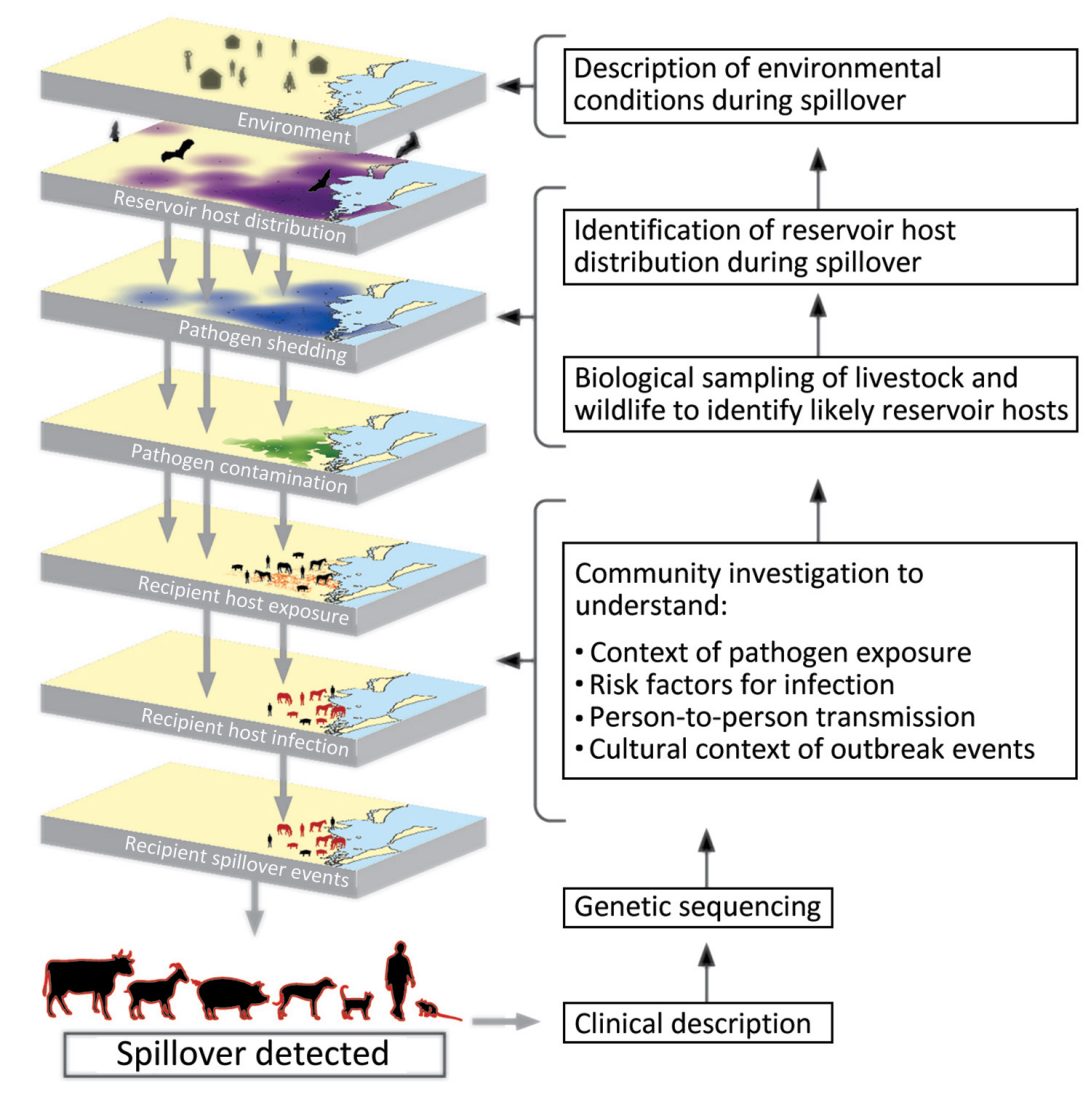
A visual guide to One Health investigations of spillovers to assist with primary pandemic prevention. One Health investigations trace spillover events backward from detection, systematically uncovering the causal chain that led to spillover. This process involves characterizing the pathogen, contexts, and risks for transmission and determining the reservoir hosts and environmental conditions that enabled the event.

One Health spillover investigations represent a crucial step in a broader continuum of actions designed to move from identifying mechanistic, proximal causes of spillover to designing and testing interventions to prevent them. This continuum from discovery to spillover prevention (Figure 2) encompasses multiple interconnected steps: discovery of the zoonotic pathogen in reservoir hosts, detection of spillover events, carrying out One Health spillover investigations, and identifying the transmission pathways and conditions that enabled spillover. The subsequent steps involve iterative research to develop, test, and deploy interventions to prevent spillovers by targeting both proximal and upstream causes. Each step informs the others, creating ongoing feedback essential for pandemic prevention.

**Figure 2:**
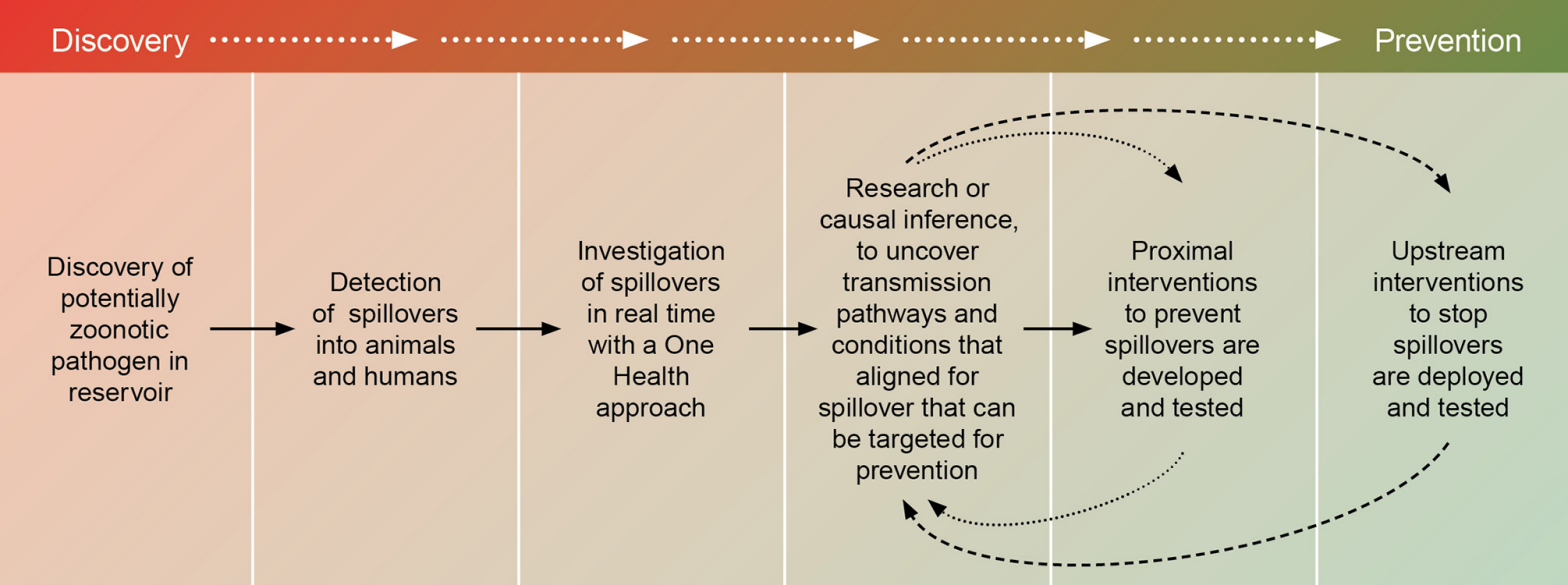
A roadmap of primary pandemic prevention through spillover investigation from discovery to the prevention of zoonotic spillover. The pathway from discovery of a zoonotic pathogen in reservoir hosts to the prevention of future spillovers often begins with the detection of spillovers in persons or domestic animals. If those detections prompt One Health investigations, followed by in-depth studies to identify the root causes of spillover, the resulting knowledge can inform the design, testing and implementation of interventions to address both proximal and distal drivers of spillover risk.

Spillover investigations are crucial for pandemic prevention, and more effort is needed to identify and study spillovers. There are multiple barriers to identifying spillovers that span global, national, and local levels. Because of those barriers, many spillovers remain undetected or unreported. At the local level, there might be insufficient resources to diagnose common causes of disease, much less rare and emerging pathogens. Even if that barrier is overcome, communities might be apprehensive about uncovering emerging pathogens because that process can lead to blame, stigmatization, and negative economic impacts. At the national level, there are political, financial, and economic threats to navigate. The reality is that spillovers are almost always negative events for governments. Spillovers are politically sensitive and sometimes not reported out of fear. Reporting of emerging pathogen outbreaks has led to severe economic outcomes for reporting countries, including travel bans or trade embargoes (*21*–*23*). Once a spillover is identified, governments might be expected to expend considerable resources for investigation and response to reduce the global pandemic risk. For governments that have threadbare budgets for combating endemic public health problems, there might be little desire to take on those additional actions. Although the numerous disincentives to spillover detection are formidable, we have much to gain by overcoming them.

When investigations of spillovers, particularly those conducted through a One Health approach, have taken place, they have yielded critical insights and even solutions to prevent future spillovers (Figure 2). For example, Hendra virus is an often-fatal virus transmitted from bats to horses and subsequently to humans in Australia. Ecologists involved in the investigations of Hendra virus spillovers noted unusual bat activity in the paddocks of affected horses. Bats were feeding on unripe figs and other foods associated with starvation avoidance. This observation prompted the researchers to hypothesize that food shortages for bats were somehow associated with spillovers. Subsequent long-term studies revealed that climate fluctuations, interacting with habitat loss, led to acute food shortages that drove bats into agricultural areas and caused them to shed Hendra virus in proximity to horses (*24*). During those investigations, researchers noted that spillovers did not occur when remnant patches of critical habitat flowered, providing food for bats. This finding suggested a potential solution: restoring critical habitats to mitigate spillovers (*24*). This example illustrates the critical role of spillover investigation and subsequent studies to understand the mechanisms underlying spillovers. When mechanisms are understood, interventions to prevent future occurrences become apparent. Restoration of critical habitat has begun, but it will take more than a decade to determine if that intervention decreases the risk for Hendra virus spillovers.

Nipah virus transmission in Bangladesh provides another excellent case study about how looking for spillovers and then conducting One Health investigations have led to major insights into proximal causes of spillover and possible targets for spillover prevention (Figure 2). The first outbreaks of Nipah virus were discovered in Bangladesh in 2001 (*25*), and after years of One Health investigations of spillovers, an understanding of the source began to form in 2005 (*26*). Epidemiologic studies identified date palm sap consumption as a key risk factor for Nipah virus infection, and social scientists studied how the sap was harvested and sold (*26*–*29*). Date palm sap is collected from trees and drunk fresh during the cool, winter months; it is a cultural delicacy (*29*). Wildlife investigations identified that bats shed virus in their urine and saliva (*30*), ecologic investigations revealed that bats drink and contaminate date palm sap as it drips into the pots (*31*), and virologic studies showed that Nipah virus is stable in date palm sap (*32*). Further studies then demonstrated that simple covers of the pots and sap stream on the tree, which were already being used by some sap collectors, would protect the sap from contact with bats (*33*,*34*).

Spillover dynamics are driven by the interaction of multiple complex systems, including infection dynamics in the reservoir hosts, their shifting population distributions, and emergent human behaviors and practices (Figure 1). Drivers span from local alterations in land use change to global climate. Investigating the underlying drivers of spillovers often requires sustained effort over years or decades (Figure 1), extending beyond the duration of individual grants, or any single person’s tenure in a particular job. However, the example of Hendra virus spillover investigations in Australia exemplifies how a strong curiosity and a commitment to understanding the mechanisms underlying spillovers can lead to the potential for ecological solutions to prevent pandemics (*24*).

In summary, we have presented evidence about how a One Health approach to spillover investigation can lead to spillover prevention by using Hendra and Nipah virus as case studies. However, those approaches are applicable to any spillover pathogen, not just viruses, and any reservoir host, not just bats. Opportunities to learn more about and prevent spillovers are frequent but often missed. We know very little about the specific spillovers that led to most of the large outbreaks or pandemics in the past 100 years, mostly because by the time investigations began, the trail was cold. For example, the origin of the 2013–2016 Ebola epidemic in West Africa was not investigated until months after its onset, leaving the initial spillover that led to that outbreak uncertain, similar to most other Ebola outbreaks (*35*,*36*). The origins of several recent spillovers remain unresolved, including how Nipah virus first spilled over to humans in Kerala, India, in 2018, 2019, and 2023 (*37*,*38*), and how H5N1 spilled over into dairy cattle in the United States (*20*). The origins of the COVID-19 pandemic are likely to remain unsolved indefinitely, because of delays in investigations. Until we dedicate ourselves to the search for and One Health investigation of spillovers, we remain vulnerable to their devastating consequences.
